# Treatment of patients with multiple organ dysfunction syndrome (MODS) with an electromagnetic field coupled to biorhythmically defined impulse configuration: the MicrocircMODS study

**DOI:** 10.1007/s00392-023-02293-2

**Published:** 2023-09-17

**Authors:** Karl Werdan, Sebastian Nuding, Diethelm Kühnert, Ramzi Kolthoum, Artjom Schott, Felix Quitter, Andreas Wienke, Daniel Sedding

**Affiliations:** 1 Department of Internal Medicine III, University Hospital Halle (Saale), Martin-Luther-University Halle-Wittenberg, Ernst-Grube-Strasse 40, 06097 Halle (Saale), Germany; 2 Hospital St. Elisabeth and St. Barbara, 06110 Halle (Saale), Germany; 3 04838 Zschepplin, Germany; 4 01277 Dresden, Germany; 5 Institute of Medical Epidemiology, Biometry and Computer Science, Martin-Luther-University Halle-Wittenberg, Magdeburger Strasse 8, 06112 Halle (Saale), Germany

**Keywords:** Electromagnetic field, Loss of haemodynamic coherence, Microcirculation, Multiple organ dysfunction syndrome (MODS), Physical Vascular Therapy BEMER^®^ (PVT)

## Abstract

**Background:**

To potentially improve impaired vasomotion of patients with multiple organ dysfunction syndrome (MODS), we tested whether an electromagnetic field of low flux density coupled with a biorhythmically defined impulse configuration (Physical Vascular Therapy BEMER^®^, PVT), in addition to standard care, is safe and feasible and might improve disturbed microcirculatory blood flow and thereby improve global haemodynamics.

**Methods:**

In a prospective, monocentric, one-arm pilot study, 10 MODS patients (APACHE II score 20–35) were included. Patients were treated, in addition to standard care, for 4 days with PVT (3 treatment periods of 8 min each day; day 1: field intensity 10.5 μT; day 2:14 μT, day 3:17.5 μT; day 4:21.0 μT). Primary endpoint was the effect of PVT on sublingual microcirculatory perfusion, documented by microvascular flow index (MFI). Patient safety, adverse events, and outcomes were documented.

**Results:**

An increase in MFI by approximately 25% paralleled 4-day PVT, with the increase starting immediately after the first PVT and lasting over the total 4-day treatment period. Concerning global haemodynamics (secondary endpoints), halving vasopressor use within 24 h, and haemodynamic stabilisation paralleled 4-day PVT with an increase in cardiac index, stroke volume index, and cardiac power index by 30%–50%. No adverse events (AEs) or serious adverse events (SAEs) were classified as causally related to the medical product (PVT) or study. Three patients died within 28 days and one patient between 28 and 180 days.

**Conclusion:**

PVT treatment was feasible and safe and could be performed without obstruction of standard patient care. An increase in microcirculatory blood flow, a rapid reduction in vasopressor use, and an improvement in global haemodynamics paralleled PVT treatment. Findings of this pilot study allowed forming a concept for a randomized trial for further proof.

**Graphical abstract:**

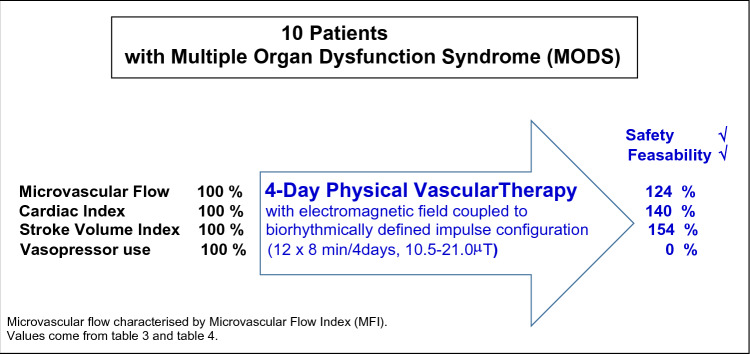

**Supplementary Information:**

The online version contains supplementary material available at 10.1007/s00392-023-02293-2.

## Introduction

Multiple organ dysfunction syndrome (MODS) [1] can be diagnosed in 90% and more of all deceased ICU patients. Cardiovascular dysfunction of cardiac or septic origin leading to shock contributes essentially to the unfavourable prognosis of MODS patients. Current standard treatment with vasopressors and cardiovascular assist devices—focussing on stabilisation of the macrocirculatory parameter “blood pressure”—yields poor results and is without proven prognostic relevance [2]. An important component of organ dysfunction in MODS is the derangement of microcirculation [3, 4], which has negative prognostic relevance [3, 5] even in cases of normalised blood pressure [6]. Microcirculatory abnormalities are frequently found—17% [7], 56% [3]—in ICU patients. However, impairment of microcirculation cannot be specifically treated with vasoactive substances because of a loss in haemodynamic coherence in MODS of cardiogenic or septic origin [6, 8, 9]. Consequently, stabilization of blood pressure by vasopressors in MODS does not guarantee improvement and normalization of impaired microcirculation and thereby organ perfusion [10]. Therefore, the search for alternative treatment concepts other than vasopressors might be worthwhile because impairment of microcirculation is principally reversible, at least in part, in patients with cardiogenic shock, and topical application of the neurotransmitter acetylcholine can completely normalize impaired sublingual microvascular perfusion [4].

A phenomenon of abnormal microcirculation is impaired vasomotion: Arteriolar vasomotion [11–14] has been propagated as the most important regulatory mechanism of local tissue and/or organ blood flow [15]. Physiologically, slow waves with oscillations of 1–5 cycles per minute can be found in arterioles from 100 μm in size (under humoral and neural control), down to precapillary arterioles (autorhythmic vasomotion), as well as in postcapillary venules [13, 15]. This vasomotion is disturbed in diabetes, hypertension, hypoperfusion, and acidosis [13], with a reduced frequency of one vasomotion per 10 min in severely ill patients [15]. While pharmacological interventions—especially in the small-calibre arterioles with their autorhythmic vasomotion—are very limited, improvement of this impaired vasomotion is claimed by an electromagnetic field of low flux density coupled to biorhythmically defined impulse configuration (Physical Vascular Therapy BEMER^®^, PVT) [15, 16].

In our MicrocircMODS study—the first PVT pilot study in MODS patients—we tested whether complementary treatment of patients with MODS for 4 days with PVT is safe and feasible and whether PVT might improve impaired vasomotion in these patients and thereby improve microcirculation by increasing the pathologically reduced vasomotion frequency. The effect of PVT on microcirculatory flow was sublingually visualised using the sidestream dark-field method (SDF), and the consequences on global haemodynamics were monitored.

## Methods

The MicrocircMODS study was an investigator-driven study sponsored by the Martin-Luther-University Halle-Wittenberg. This study is listed in the German Clinical Trials Registry under DRKS-ID: DRKS00006741 (Sept. 12, 2014).

### Study design

MicrocircMODS was a prospective, monocentric, one-arm pilot study. In ten MODS patients, the effects of a complementary four-day whole-body treatment (three 8-min periods per day) with PVT were investigated with respect to changes in sublingual microcirculation, global haemodynamics, MODS, prognosis, and safety.

The **primary endpoint** was sublingual microcirculation during the four-day treatment period, characterised by total vessel density (TVD all, TVD small: vessels ≤ 20 μm in diameter, TVD non-small: vessels > 20 μm in diameter), and microcirculatory flow index (MFI).

The **secondary endpoints** were as follows:

- Global haemodynamics: cardiac index (CI), cardiac power index (CPI), afterload-related cardiac performance (ACP) [17], vasopressor use quantified by vasopressor score (VS) [18], heart rate (HR), and heart rate variability (HRV) [19]. HRV was characterized by the parameters SDNN (standard deviation of the NN intervals), SDANN (standard deviation of the averages of the NN intervals in all five minute segments), pNN50 (proportion of differences between adjacent NN intervals exceeding 50 ms), rMSSD (the* s*quare root of the mean of the sum of the squares of differences between adjacent NN intervals), high-frequency power (HF), LF (low-frequency power), VLF (*v*ery-low-frequency power), and LF/HF (ratio of low- versus high-frequency power). In a post hoc analysis, stroke volume index (SVI) was also calculated.
- The severity of MODS, as measured by the APACHE II score [20], by serum lactate levels as measured for tissue hypoperfusion, and by coagulation disturbances as quantified by the disseminated intravascular coagulation (DIC) score [21].
- ICU-, hospital-, 28-day- and 180-day mortality.
- For safety analysis, adverse events (AE), serious AE (SAE), adverse device effects (ADE), serious ADE (SADE), and disease-related events (DRE) were monitored according to international nomenclature.

Data monitoring, patient follow-up and study data analysis were performed by the Coordination Centre for Clinical Studies Halle of the Medical Faculty of the Martin-Luther-University Halle-Wittenberg.

SAS version 9.4 (SAS Institute Inc., NC, USA) and SPSS version 26 (IBM Deutschland GmbH, Ehningen, Germany) were used for analysis. Data from this pilot study were analysed in a descriptive manner; no statistical tests were carried out, and no *p* values were calculated. If not stated otherwise, metric data are presented as medians with interquartile ranges (IQR).

An independent data monitoring committee (see the appendix) accompanied the pilot study. After including five of the planned ten study patients, the patient data were presented to the DSMB members; the DSMB members decided that the study could be continued without any restrictions.

### Patients

The study was performed in the 12-bed medical ICU of the Department of Internal Medicine III at University Hospital Halle (Saale), Germany. Enrolment of the ten patients with MODS of either cardiac or septic origin took place between November 2015 and April 2018. This study ended in October 2018.

#### Inclusion criteria

(a) Having MODS, diagnosed within the previous 24 h and characterised by an APACHE II score of ≥ 20 and ≤ 35; (b) study-independent indication for invasive hemodynamic monitoring; and (c) written informed consent by the patient, the health care proxy, or the provisional supervisor.

#### Exclusion criteria

(a) Age < 18 years; (b) pregnancy or lactation; (c) malignant hyperthermia; (d) cell transplantation, bone marrow transplantation, stem cell transplantation, or organ transplantation; (e) participation in other trials (contemporaneously or within the last three months), and (f) withdrawal of life-sustaining measures had to be taken into account according to the will of the patient.

#### Historical control group

Post hoc, a historical control group was chosen to compare macro-haemodynamic parameters of PVT-treated MicrocircMODS study patients with MODS patients from the MOD*I*_f_Y trial (NCT01186783; “Reducing elevated heart rate in patients with multiple organ dysfunction syndrome with the *I*_f_ [funny channel current] inhibitor ivabradine”) [22], which had been conducted in our ICU from 2010 to 2012. Study patients in the MOD*I*_f_Y trial had MODS diagnosed within the previous 24 h of either cardiogenic or septic origin, with an APACHE II score of ≥ 20 and sinus rhythm of ≥ 90 beats per min. Matched pairs were chosen according to the baseline heart rate and baseline APACHE II score.

### Physical Vascular Therapy BEMER^®^ (PVT)

**Fig. 1:**
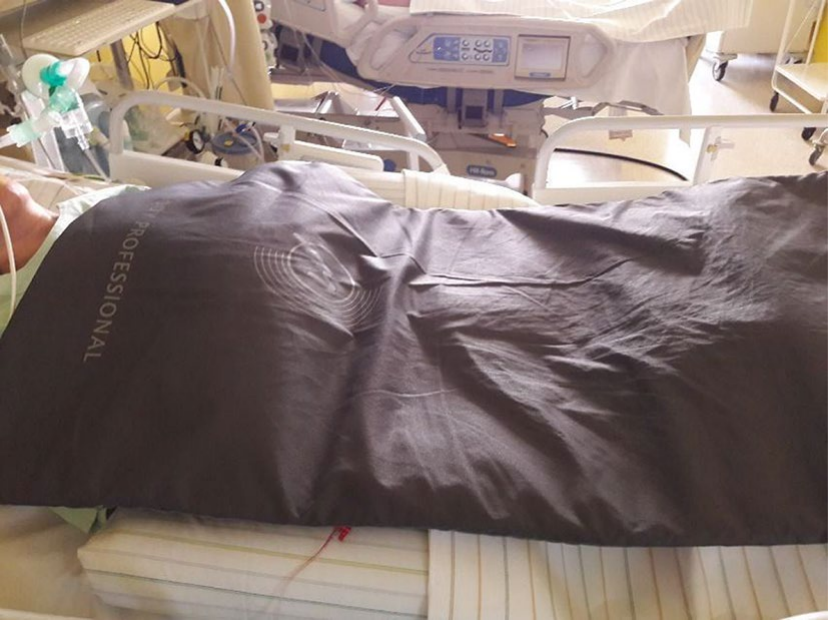
Physical Vascular Therapy BEMER^®^—Placement of the mat “B.BODY Pro” for whole body treatment of the study patient. For further explanation, see “Methods”

**Fig. 2:**
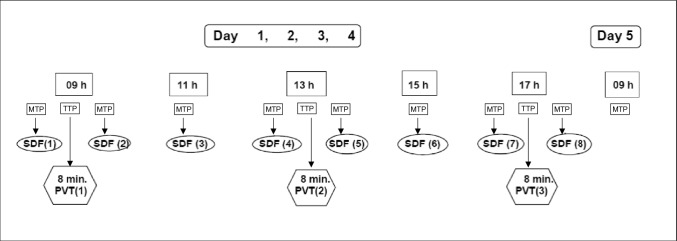
Study concept of the MicrocircMODS Study. For further explanation, see Table 1. *MTP* measurement time point; *PVT* Physical Vascular Therapy BEMER^®^ (field intensity**:** day 1: 10.5 μT**,** day 2: 14.0 μT**,** day 3: 17.5 μT**,** day 4: 21.0 μT); *SDF* sublingual sidestream dark-field monitoring (Microscan^®^); *TTP* therapy time point

In addition to the standard treatment, complementary PVT was performed using the BEMER PRO Set (console unit B.BOX plus applicators; BEMER International AG Triesen, Liechtenstein): A specific, biorhythmically defined stimulus signal is used to transfer energy to the “resonator” small-calibre arterioles by a weak electromagnetic field (i.e. flux density ≤ 100 μTesla). The electromagnetic field was applied to the patient as a whole-body treatment by a mat, 180 × 60 × 2 cm (B.BODY Pro), placed on the patient like a blanket from the neck to the toes (Fig. 1). PVT was given on four consecutive days, three times a day, for eight minutes each, with controlled flux densities of 10.5 μT on day 1, 14,0 μT on day 2, 17.5 μT on day 3, and 21.0 μT on day 4 (Fig. 2). For chronobiological reasons, treatment and monitoring were started each day at 9.00 a.m. (Fig. 2, Table 1). Per-protocol patients were defined as those who received at least 10 of the 12 planned PVTs.

**Table 1:** Study plan of the MicrocircMODS study

Day	Day 1–4											Day 5
Time	Before	09 h	After	11 h	Before	13 h	After	15 h	before	17 h	After	09 h
Measurement Time Point (MTP) on day (D) 1–5	D 1: 1		D 1: 2:	D1: 3	D1: 4		D1: 5	D1: 6	D1: 7		D1: 8	33
	D 2: 9		D 2: 10	D2: 11	D2: 12		D2: 13	D2: 14	D2: 15		D2: 16	
	D 3: 17		D 3: 18	D3: 19	D3: 20		D3: 21	D3: 22	D3: 23		D3: 24	
	D 4: 25		D 4: 26	D4: 27	D4: 28		D4: 29	D4:30	D4: 31		D4: 32	
PVT (8 min each)		x				x				x		∅
SDF	x		x	x	x		x	x	x		x	∅
Data set (Fig. 2):												
ECG												
ECG	x		x						x		x	x
Haemodynamic data set												
HR (1/min)	x		x	x	x		x	x	x		x	x
SAP (mm Hg)	x		x	x	x		x	x	x		x	x
DAP (mm Hg)	x		x	x	x		x	x	x		x	x
MAP (mm Hg)	x		x	x	x		x	x	x		x	x
CVP (mm Hg)	x		x	x	x		x	x	x		x	x
HRV	x		x	x	x		x	x	x		x	x
CO (l/min)	x		x	x	x		x	x	x		x	x
Ventilatory data set												
Mode of ventilation	x											x
Laboratory data set												
Leucocytes (× 10^9^/l)	x											x
Hb (mmol/l)	x											x
Platelets (× 10^9^/l)	x											x
Serum-K^+^ (mmol/l)	x											x
Serum-creatinine (μmol/l)	x											x
Serum-urea-N (μmol/l)	x											x
Diuresis (ml/h)	Hourly from 9 a.m. to 5 p.m											x
Lactate (mmol/l)	x										x	x
Vasopressors/Inotropes (mean last 12 h)												
Norepinephrine	x											x
Epinephrine	x											x
Dobutamine	x											x
Dopamine	x											x
Scores & body temperature												
APACHE II Score	x											x
Coagulation Score	x											x
Body temperature (°C)	x		x	x	x		x	x	x		x	x
Safety data set												
AE, SAE, DRE, ADE, SADE	x			x		x			x	x		x

Data sets (Fig. 2) were documented at the times indicated, including ECG, haemodynamic, ventilatory and laboratory data sets, vasopressor score and body temperature, scores and safety data sets

Vasopressor score [18] was calculated in 12 h intervals before, during and after PVT

*ADE* adverse device event, *AE* adverse event, *CO* cardiac output, *CVP* central venous pressure, *DAP* diastolic arterial pressure, *DRE* Disease-related event, *ECG* electrocardiogram, *Hb* haemoglobin, *HR* heart rate, *HRV* heart rate variability, *MAP* mean arterial pressure, *PVT* Physical Vascular Therapy BEMER^®^, *SADE* serious adverse device effect, *SAE* serious adverse event, *SAP* systolic arterial pressure, *SDF* Sidestream dark-field

PVT [15] is used in complementary medicine for several indications. This concept is described as follows (based on the information provided by the manufacturer BEMER Int. AG): PVT is based on a pulsed sinus wave, which consists of a string of single impulses. These start with a base intensity and are continuously increasing over the course of 16 repetitions from 3.5 µT up to 100 µT. The signal is divided into two major frequency bands: 10 Hz and 30 Hz. The duration of a signal sequence is 120 s in total, starting with 83 s of the 30 Hz basic signal, followed by 31 s of the 10 Hz signal. There is a 3-s pause between each frequency change. A pulsed signal can be added to the basic signal sequence as an option. This signal increases the flux density from 100 to 150% and is repeated every 20 s during the course of the basic signal. The "plus" signal seems to have an additional impact on the impaired vasomotion.

### Monitoring of the microcirculation

The sublingual microvascular network was studied using a sidestream dark-field microcirculation camera (Microscan, Microvision Medical, Amsterdam, The Netherlands), according to a standardized scoring system [23]: green light emitted by the camera and absorbed by red blood cells is used as contrast agent to visualize sublingual blood flow in the microvessels: a handheld device (Microscan, Microvision Medical, Amsterdam, The Netherlands) was carefully applied on the sublingual mucosa to obtain two-dimensional video loops of sublingual microcirculatory blood flow. The saliva was carefully removed. Five 10-s recordings from different areas of the sublingual mucosa were taken in a defined order without any movement artefacts using a microscopic camera connected to a laptop computer. Recordings hampered by saliva or movement artefacts were discarded. Eight measurements were taken per day: one measurement each immediately before and after the three PVTs a day, and two additional measurements between PVT 1 and PVT 2 and between PVT 2 and PVT 3 (Fig. 2). Each measurement consisted of five 10-s recordings from different areas of the sublingual mucosa.

Sequences were analysed offline in a blinded fashion by trained study investigators in cooperation with an experienced independent colleague (F. Quitter, University Hospital Carl Gustav Carus, Dresden, Germany), who was not involved in the MicrocircMODS study, using analytical software (Automated Vascular Analysis, AVA 3.0, Microvision Medical) and manual corrections. Study endpoints (see “study design”) were—in agreement with the minimum dataset of microvascular variables per cardiogenic shock [24]—vessel density (in mm/mm^2^) as total vessel density (TVD), total vessel density of small (≤ 20 μm in diameter) vessels (TVD small) and total vessel density of non-small (> 20 μm in diameter) vessels (TVD non-small) as well as the microvascular flow index (MFI). The microvascular flow index (MFI) is based on determination of the predominant type of flow in four quadrants of the image [23]: Flow is characterised as “absent” (0 points), “intermittent” (1 point), “sluggish” (2 points) or “normal” (3 points). The values of the four quadrants were averaged to obtain the MFI.

## Results

### Patient characteristics

**Fig. 3:**
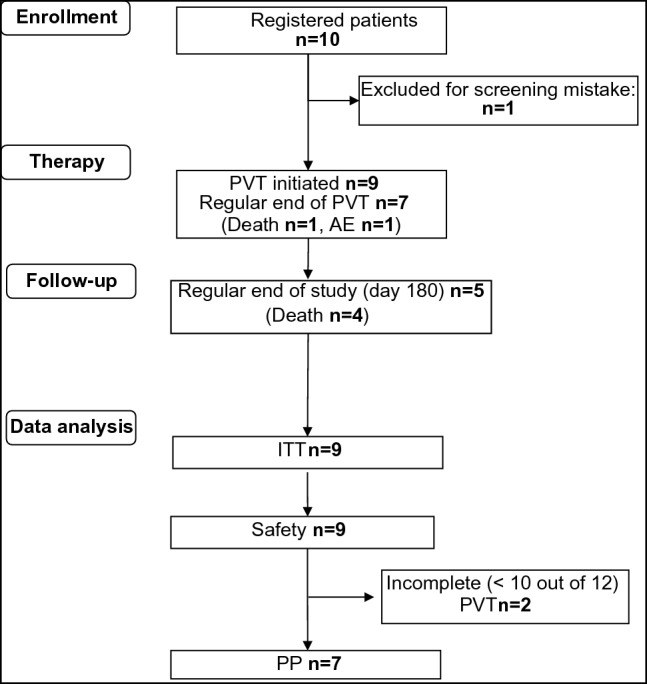
Flow sheet of the MicrocircMODS study. Patient no. 6 was screened and registered but could not be included in the study because of a screening mistake (withdrawal of invasive cardiac output measurement in the interval from inclusion of the patient/reporting to the coordination centre for clinical trials and starting Physical Vascular Therapy BEMER^®^ (PVT)). Patient nos. 2, 3, 4, 5, 7, 9 and 10 completed all 12 PVTs (4 days, three times a day). Patient number 1 received two PVTs on day 1, three PVTs on day 2 and one PVT on day 3; thereafter, patient had to be transferred to the surgery department for a disease-related emergency operation. Patient number 8 received three PVTs on day 1 and day 2; before the first PVT was intended to be given on day 3, the patient died from MODS and septic shock. *IT* Intention-to-Treat Group, *PP* Per-Protocol Group, *PVT* Physical Vascular Therapy BEMER^®^

Figure 3 shows the flow sheet of the MicrocircMODS trial. Of the ten patients registered as study patients, seven patients completed the therapy period, including ≥ 10 out of 12 PVTs (3 PVTs each on days 1–4), and nine patients were included in the intention-to-treat group (ITT-group) as well as in the safety analysis. Table 2 shows the characteristics of the study patients—with the youngest patient being 54 years old and the oldest 89 years old—as well as the characteristics of the historical control patients from the MOD*I*_f_Y trial [22]. Of importance for comparison, both groups had a median value of the APACHE II score [20] of 32, indicating a similar grade of severity of disease.

**Table 2:** Patient characteristics of the MicrocircMODS study and the historical control group ((MOD*I*_*f*_Y trial; [22])

	MicrocircMODS	Historical control (MOD*I*_*f*_Y trial) [22]
Number of patients (ITT)	9	9
Male/female	4/5	7/2
Age [years] (median (IQR))	65 (57, 78)	73 (54, 78)
Height [cm] (median (IQR))	165 (165, 175)	175 (165, 180)
Weight [kg] (median (IQR))	99.5 (70.0, 107.0)	85 (80, 92)
BMI [kg/m^2^] (median (IQR))	32.49 (25.71, 36.73)	27,76 (26.23, 31.25)
BSA [m^2^] (median (IQR))	2.061 (1.771, 2.250)	2.05 (1.83, 2.10)
Origin of MODS	Septic 5 Cardiac 4	Non-coronary 7 Coronary 2
Initial APACHE II score (median (IQR))	32 (27, 33)	32 (27, 37)
Initial DIC score (median (IQR))	3.0 (2.0, 5.0)	n.d.
Comorbidities		n.d.
Coronary heart disease	4	
Chronic obstructive pulmonary disease	3	
Heart failure	2	
Chronic kidney disease	0	
Stroke	0	

In the MicrocircMODS study, MODS has been classified either of septic or of non-septic origin; in the MOD*I*_*f*_Y trial, MODS has been classified either of coronary or of non-coronary aetiology [22]

*BMI* body mass index, *BSA* body surface area, *DIC* disseminated intravascular coagulation, *IQR* interquartile range, *ITT* intention-to-treat group, *MODS* multiple organ dysfunction syndrome, *n.d.* not determined

### Physical vascular therapy and microcirculation

**Fig. 4:**
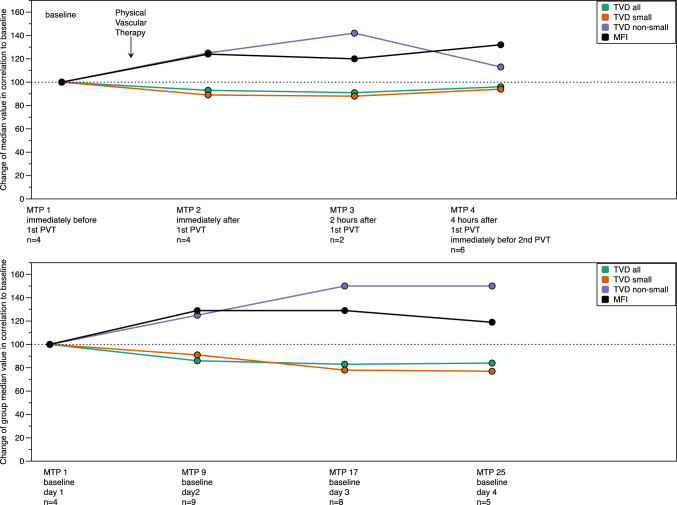
Effect of Physical Vascular Therapy BEMER^®^ (PVT) in MODS patients on sublingual microcirculation—Follow-up of first 8-min-treatment episode on day 1 (upper graph) and during the 4-day treatment (lower graph). Data are given in the ordinate as relative median values for total vascular density (TVD) (“all” = all vessels”; “small” = vessels ≤ 20 μm in diameter; “non-small = vessels > 20 μm in diameter) and microvascular flow index (MFI) (see inset). Baseline value on day 1 before PVT is set as 100%. Relative median values given in percent refer to the value of the specific measurement time point (MTP) in relation to baseline value on day 1 before 1st PVT. For median values and interquartile range (IQR) values, see Table 3. For specification of “Measurement Time Points” (MTP) see Table 1

Immediately after the first 8-min PVT (10.5 μT) on day 1, higher values for microvascular flow index (MFI) as well as for non-small vessel density (TVD non-small) by 24% and 25%, respectively, were seen (Fig. 4, upper graph; Table 3). These higher values were sustained over the entire PVT 4-day period (Fig. 4, lower graph; Table 3). Despite increasing PVT flux densities from day to day, no further increase of MFI and TVD “non-small” was observed during follow-up (Fig. 4, lower graph; Table 3). No increase during PVTs, but even fluctuating lower levels down to 77% of the initial value, were seen in the microcirculation parameters TVD all and TVD small (Fig. 4 upper and lower graph; Table 3).

**Table 3:** Sublingual microcirculation in study patients during the 4-day treatment with Physical Vascular Therapy BEMER^®^ (PVT)

Indicator	Value	Day 1	Day 1	Day 1	Day 1	Day 2	Day 3	Day 4	Day 4
Measurement		Baseline immediately before 1st PVT	Immediately after 1st PVT	2 h after 1st PVT	4 h after 1st PVT, immediately before 2nd PVT	Baseline before 1st PVT	Baseline before 1st PVT	Baseline before 1st PVT	Immediately after 3rd PVT
Time Point (MTP) (Table 1)		MTP 1	MTP 2	MTP 3	MTP 4	MTP 9	MTP 17	MTP 25	MTP 32
TVD all [mm/mm^2^]	*N*	4	4	2	6	9	8	5	5
	Absolute Median (IQR)	18.3 (17.0–19.0)	17.1 (16.4–18.1)	16.7 (15.4–18.00)	17.9 (17.1–18.8)	15.8 (15.2–18.1)	15.1 (14.5–16.5)	15.3 (15.3–19.2)	16.2 (15.9–16.3)
	Relative Median	100%	93%	91%	98%	86%	83%	84%	89%
TVD small [mm/mm^2^]	*N*	4	4	2	6	9	8	5	5
	Absolute Median (IQR)	17.1 (15.8–17.8)	15.3 (15.1–16.1)	15.0 (12.8–17.1)	16.0 (15.5–17.4)	15.5 (13.3–17.5)	13.3 (12.3–14.6)	13.1 (11.9–17.9)	14.7 (13.8–14.9)
	Relative Median	100%	90%	88%	94%	91%	78%	77%	86%
TVD non-small [mm/mm^2^]	*N*	4	4	2	6	9	8	5	5
	Absolute Median (IQR)	1.2 (1.2–1.3)	1.50 (1.4–2.0)	1.7 (0.9–2.5)	1.4 (0.8–1.9)	1.5 (0.9–1.9)	1.9 (1.3–2.5)	1.8 (1.3–2.1)	1.4 (1.3–2.3)
	Relative Median	100%	125%	142%	113%	125%	150%	150%	117%
MFI	*N*	4	4	2	6	9	8	5	5
	Absolute Median (IQR)	2.1 (1.4–2.4)	2.6 (2.3–2.7)	2.5 (2.4–2.5)	2.7 (2.4–2.7)	2.7 (2.4–2.8)	2.7 (2.3–2.8)	2.5 (2.3–2.7)	2.6 (2.3–2.6)
	Relative Median	100%	124%	119%	129%	129%	129%	119%	124%

Total vascular density (TVD all, TVD small, TVD non-small) and microvascular flow index (MFI) results are presented at the measurement time points (MTPs) indicated. For MTPs, see Table 1. “Absolute Median” refers to the median value of the recorded parameter. “Relative Median” refers to the value of the specific day in relation to baseline median of day 1 and is given in percent

*N* Number of patients measured

### Physical vascular therapy and macrocirculation

**Fig. 5.**
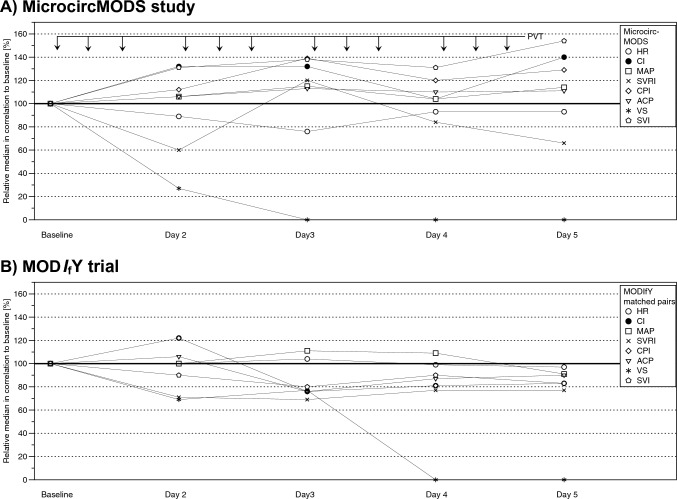
4-day Physical Vascular Therapy BEMER^®^ (PVT; day 1–day 4; 3 treatments per day; ↓) and global haemodynamics of MicrocircMODS study patients (**A**)—Comparison with the data from historical control MODS patients (MOD*I*_f_Y trial [22]) (**B**). **A** Ordinate: Haemodynamic data (see inset) are given as relative median values, with the value before the first PVT on day 1 (“Baseline”) set as 100%. Abscissa: “Baseline” = Day 1 Baseline (09,00 a.m.) immediately before 1st PVT (MTP 1); “Day 2” = Day 2 Baseline (09.00 a.m.) before 1st PVT (MTP 9); “Day 3” = Day 3 Baseline (09.00 a.m.) before 1st PVT (MTP 17); “Day 4” = Day 4 Baseline (09.00 a.m.) before 1st PVT (MTP 25); “Day 5” = Day 5 Baseline (09.00 a.m), first measurement after last (3rd) PVT on day 4) (MTP 32). **B** Ordinate: Haemodynamic data (see inset) are given as relative median values, with the value on day 1 (“Baseline”) set as 100%. Abscissa: Measurements during the first five days after inclusion of MODS patients in the MOD*I*_f_Y trial [22]. *ACP* Afterload-related Cardiac Performance, *CPI* Cardiac Power Index, *CI* Cardiac Index, *HR* Heart Rate, *MAP* Mean Arterial Pressure, *MTP* Measurement Time Point (see Table 1), *PVT* Physical Vascular Therapy BEMER^®^, *SVI* Stroke Volume Index, *SVRI* Systemic Vascular Resistance Index, *VS* Vasopressor Score

Figure 5A and Table 4 (Part a) present the macrocirculatory data of the patients before the first PVT on day 1 (“baseline”), during PVT (“Day 2–Day 4”) and the following day after the last PVT on day 4 (“Day 5”).

**Table 4:** Time course of global haemodynamics in patients of the MicrocircMODS study before, during and after the 4-day Physical Vascular Therapy BEMER^®^ (PVT) (Part a) and comparison of data with those of historical control patients (matched pairs of the MOD*I*_f_Y trial [22] (Part 4b)

		Part a: PVT (MicrocircMODS study)					Part b: Control (matched pairs from MOD*I*_f_Y trial)				
Indicator	Value	Day1	Day 2	Day 3	Day 4	Day 5	Day 1	Day 2	Day 3	Day 4	Day 5
		Baseline before 1st PVT on day 1	Baseline before 1st PVT on day 2	Baseline before 1st PVT on day 3	Baseline before 1st PVT on day 4	Baseline	Baseline				
		MTP 1	MTP 9	MTP 17	MTP 25	MTP 33					
APACHE II Score	*N*	9	9	9	8	8	9	9	9	9	8
	Absolute Median (IQR)	32 (27–33)	32 (27–34)	29 (24–32)	23 (18–26)	25 (20–30)	32 (27–38)	30 (26–31)	30 (25–32)	28 (24–34)	28 (23–32)
	Relative Median	100	100	91	72	78	100	94	94	88	88
HR [1/min]	*N*	8	9	7	7	6	9	9	9	9	9
	Absolute Median (IQR)	92 (73–101)	82 (78–97)	70 (66–97)	86 (69–95)	86 (69–95)	90 (83–96)	90 (84–92)	94 (89–97)	89 (83–95)	87 (78–90)
	Relative Median	100	89	76	93	93	100	100	104	99	97
CI [l/min/m^2^]	*N*	7	8	8	7	3	5	5	5	7	6
	Absolute Median (IQR)	2.5 (2.1–3.1)	3.3 (2.7–3.6)	3.3 (2.6–3.7)	2.6 (2.2–3.3)	3.5 (3.3–4.1)	3.5 (2.3–3.9)	3.7 (2.3–3.8)	2.4 (2.1–5.0)	3.0 (2.2–4.8)	2.8 (2.3–3.8)
	Relative Median	100	132	132	104	140	100	106	69	86	80
SVI [ml/m^2^]	*N*	7	8	7	7	3	5	5	5	7	6
	Absolute Median (IQR)	26 (20–44)	34 (28–46)	36 (29–53)	34 (26–43)	40 (31–48)	40 (26–42)	36 (34–45)	32 (25–53)	36 (26–54)	33 (30–43)
	Relative Median	100	131	138	131	154	100	90	80	90	83
MAP [mmHg]	*N*	8	9	7	7	6	9	9	9	9	9
	Absolute Median (IQR)	71 (63–81)	75 (61–75)	82 (76–87)	74 (67–93)	81 (70–95)	76 (68–85)	76 (74–91)	84 (74–85)	83 (67–102)	69 (57–81)
	Relative Median	100	106	115	104	114	100	100	111	109	91
SVRI [dynxcm^−5^xsxm^−2^]	*N*	7	8	7	7	3	3	5	5	6	6
	Absolute Median (IQR)	559 (266–1163)	335 (266–591)	673 (298–910)	467 (334–1013)	369 (217–446)	464 (319–526)	331 (305–361)	322 (267–733)	355 (329–635)	355 (314–779)
	Relative Median	100	60	120	84	66	100	71	69	77	77
CPI [Wxm^−2^]	*N*	7	8	7	7	3	5	5	5	7	6
	Absolute Median (IQR)	0.41 (0.34–0.49)	0.46 (0.39–0.62)	0.57 (0.40–0.67)	0.49 (0.36–0.54)	0.53 (0.49–1.04)	0.59 (0.39–0.64)	0.72 (0.39–0.74)	0.45 (0.40–0.93)	0.48 (0.35–0.90)	0.49 (0.34–0.59)
	Relative Median	100	112	139	120	129	100	122	76	81	83
ACP [%]	*N*	7	8	7	7	3	3	5	5	6	6
	Absolute Median (IQR)	70 (57–79)	74 (64–89)	79 (65–93)	77 (63–81)	78 (77–132)	86 (78–93)	91 (55–94)	65 (54–110)	75 (67–112)	77 (77–84)
	Relative Median	100	106	113	110	111	100	106	76	87	90
VS	*N*	9	9	8	7	6	9	9	9	9	9
	Absolute Median (IQR)	30 (15–35)	8 (2–26)	0 (0–12)	0 (0–14)	0 (0–14)	13 (1–38)	9 (1–26)	10 (0–52)	0 (0–33)	0 (0–13)
	Relative Median	100	27	0	0	0	100	69	77	0	0

Haemodynamic data are presented from MODS patients of the MicrocircMODS study (Part a) and from MODS patients (matched pairs) of the MOD*I*_f_Y trial [22] as historical controls (Part b). Results of the MicrocircMODS study (Part a) are presented as values collected at 9:00 a.m. before starting PVTs of the days 1–4 (MTPs 1, 9, 17, 25) and 9.00 a.m. of day 5 (MTP 33). For MTPs see Table 1. Values are given as absolute medians with interquartile ranges (IQR) and as relative medians (%), with the median value on day 1 defined as 100%

*ACP* Afterload-related cardiac performance, *CI* cardiac index, *CPI* cardiac power index, *HR* heart rate, *IQR* interquartile range, *MAP* mean arterial pressure, *MTP* measurement time point, *N* number of patients measured, *PVT* Physical Vascular Therapy BEMER^®^, *SVI* stroke volume index, *SVRI* systemic vascular resistance index, *VS* Vasopressor Score [18], given as mean last 12 h

In a descriptive manner, stabilised global haemodynamics parallels the four-day PVT period, as seen by an increase in cardiac index (CI) by 1.0 l × min × m^−2^ (∆ + 40%), mainly mediated by a rise in stroke volume index (SVI) from 26 ml × m^−2^ before to 40 ml × m^−2^ after PVT, despite a reduction/termination of norepinephrine/dobutamine therapy (Table 5), as quantified by a reduction of the Vasopressor Score [18] from 30 units before PVT to 8 units 24 h and to 0 unit 48 h resp. after starting PVT (Table 4 Part a; Fig. 5A). Haemodynamic improvement was accompanied by an increase in mean arterial pressure (MAP) by 10 mm Hg (∆ + 14%) and a lowering of systemic vascular resistance index (SVRI) by 190 dyn × cm^−5^ × s × m^−2^ (∆ -34%) (Fig. 5A and Table 4 (Part a)).

**Table 5:** Time course of serum lactate levels and of haemodynamic support with norepinephrine and dobutamine in patients of the MicrocircMODS study before, during and after the 4-day Physical Vascular Therapy BEMER^®^ (PVT)

		PVT (MicrocircMODS study)				
Indicator	Value	Day1	Day 2	Day 3	Day 4	Day 5
		Baseline before 1st PVT on day 1	Baseline before 1st PVT on day 2	Baseline before 1st PVT on day 3	Baseline before 1st PVT on day 4	Baseline
		MTP 1	MTP 9	MTP 17	MTP 25	MTP 33
Lactate [mmol/l]	*N*	9	9	8	7	6
	Absolute Median (IQR)	2.2 (1.4–2.9)	0.9 (0.7–1.9)	1.3 (0.8–1.9)	0.8 (0.4–1.6)	0.9 (0.6–1.6)
	Relative Median	100%	41%	59%	36%	41%
Norepinephrine [mg/12 h]	*N*	9	9	8	7	6
	Absolute Median (IQR)	12.60 (6.78–14.17)	1.98 (1.51–12.07)	0.08 (0–6.29)	0 (0–7.06)	0 (0–7.03)
	Relative Median	100%	16%	0.6%	0%	0%
Dobutamine [mg/12 h]	*N*	9	9	8	7	6
	Absolute Median (IQR)	106.4 (0–216.0)	67.5 (0–121.0)	0 (0–0)	0 (0–0)	0 (0–0)
	Relative Median	100	64%	0%	0%	0%

Values are given as absolute medians with interquartile ranges (IQR) and as relative medians (%), with the median value on day 1 defined as 100%. Norepinephrine and dobutamine treatment are given as cumulative doses within the 12-h period before baseline of the respective day

*IQR* interquartile range, *MTP* measurement time point (see Table 1), *N* numbers of patients measured, *PVT* Physical Vascular Therapy BEMER^®^

Two hours after the first PVT on day 1, higher values were seen for CI by more than 0.5 l x min^−1^ × m^−2^ (∆ + 20%) (Supplemental Table S1), cardiac power index (CPI) by 29% and load-independent afterload-related cardiac performance (ACP [17]) by 11%, lasting for the complete 4-day treatment period. Concerning lactate serum levels, there was a fall from 2.20 mmol × l^−1^ (median) at baseline (day 1) before first PVT to about 1 mmol × l^−1^ (median) during and till the end of PVT (Table 5).

In MODS patients under standard care, the haemodynamic stabilisation of the study patients seen during PVT (Fig. 5A; Table 4 Part a) is rather unusual: in a historical control group of matched pairs of MODS patients under standard care (Fig. 5B; Table 4 Part b), no increase in CI was seen within the initial four-day period, and MAP did not stabilise, despite more intense and longer vasopressor use than in the MicrocircMODS patient group.

We also investigated the effects of PVT on cardiac autonomic function, as measured by parameters of heart rate variability (HRV) (see “Methods”). These findings (Supplemental Table S2) are compatible with the abnormal HRV spectrum in MODS [19]; however, no clear effect of PVT on HRV was observed.

### Multiple organ dysfunction syndrome and coagulation disorder

Serial scoring by the APACHE II score, a measure of MODS, showed a median decrease of 7 points (from 32 to 25 points) during the four-day PVT period (Table 4 Part a). Matched pairs of the post hoc historical control of the MOD*I*_f_Y trial (Table 4 Part b) yielded a decrease in the median APACHE II score by four points (from 32 to 28 points).

Coagulation disorders can represent considerable organ dysfunction in patients with MODS. In our patients, no overt DIC was observed; therefore, the ISTH overt DIC score [21] was relatively low and did not change significantly during PVT; median values were 3.0, 3.0, 2.5, 3.0, and 3.0 on the baseline of day 1 (before the first PVT) and on days 2, 3, 4, and 5, respectively.

### Safety analysis

Three of the four AEs—patient 1, skin necrosis; patient 2, decubital ulcer; patient 7, lethal pulseless electrical activity; and patient 8, lethal septic shock—were classified as SAEs: patients 1, 7, and 8. None of the AEs or SAEs were classified as causally related to the investigated medical product (Physical Vascular Therapy BEMER^®^, PVT) or to the study; therefore, none of the events were classified as SADE.

Three of the study patients died in the ICU/within 28 days: patient 2 died due to septic shock on day 13, patient 7 died due to cardiogenic shock on day 6, and patient 8 died due to MODS and septic shock on day 3 during PVT. After discharge from the hospital, another patient (patient 5) died on day 59 within the 180-day study period, because of mesenterial ischaemia. In total, four of the nine patients died within the 180-day study period. None of the deaths were classified as causally related to the investigated medical product (Physical Vascular Therapy BEMER^®^ (PVT)), or to the study.

## Discussion

### Physical Vascular Therapy and microcirculation

In our MicrocircMODS pilot study, we attempted to bypass the loss of haemodynamic coherence and directly improve impaired microcirculation. For this, we applied Physical Vascular Therapy BEMER^®^ (PVT) in addition to the standard treatment. PVT combines pulsed electromagnetic field therapy (PEMF) with a specific biorhythmically defined stimulus for vasomotion (see also “Methods”). Physical Vascular Therapy BEMER^®^ (PVT) has been described as superior to five electromagnetic field therapy devices without a specific biorhythmically defined stimulus for vasomotion and to placebo in stimulating impaired microcirculation—cell-perfused microvessels, venular flow, venular oxygen saturation, and arteriolar vasomotion—in the subcutis of study participants [25]. A positive effect of PVT on subcutaneous vasomotion and microcirculatory function has been observed in patients with diabetic polyneuropathy [26] and in rehabilitation patients [16, 27]. In young healthy volunteers, however, PVT over three weeks did not stimulate microcirculatory flow, as measured by laser Doppler placed on anterior lateral thigh, over a period of three weeks [28].

To monitor the effects of PVT on the sublingual microcirculation in our patients, we used the sidestream dark-field (SDF) method in a recommended standardised approach [23, 24], which is accepted as a useful method to characterise microcirculatory abnormalities in ICU patients in clinical studies [3, 6]. In our MicrocircMODS patients, PVT was paralleled by an increase in the microvascular flow index (MFI) (Table 3, Fig. 4). Before starting PVT, microvascular flow was clearly abnormal, according to a median MFI of 2.1, which is within the same range as the MFI values of patients with cardiogenic shock (MFI = 2.3 [6], MFI = 2.1 [29]) and of patients with MODS (MFI = 2.6 [9]). During PVT, MFI increased in our MicrocircMODS patients from 2.1 to values of 2.5–2.7, thereby leaving the range under the cut-off of 2.6, which represents a threshold below which alterations in microcirculatory flow can be considered clinically relevant [3, 24]. An increase in MFI by 24% (Table 3) was observed immediately after the first 8-min PVT on day 1 (Fig. 4, upper graph) at the lowest electromagnetic field intensity (10.5 μT) applied (see legend to Fig. 2).

Density network of small vessels (TVD small) was approximately 17 mm/mm^2^ (median) before PVT and did not increase during the 4-day PVT period, but actually was 14% less after the four-day PVT period (Table 3). The TVD small values of our patients with a median APACHE II score of 32 were somewhat lower than the TVD small values (mean 20 mm/mm^2^) reported for a mixed group of critically ill ICU patients in the MicroDAIMON study with a mean APACHE II score of 16 [3], but higher than the TVD values (mean 11 mm/mm^2^) in patients with MODS following traumatic shock [9]. Total vessel density (TVD) showed a course similar to that of TVD small (Table 3; Fig. 4A, B). In contrast, an increase in the TVD non-small vessels by 13%–50% was observed during the PVT period (Table 3; Fig. 4A, B). This group of vessels with a diameter between 20 and 100 μm is less well characterized than microvessels (TVD small) with a diameter of < 20 μm, the latter containing arterioles, capillaries (< 10 μm), and venules [24]. As these non-small vessels might be sensitive to transmitter substances like catecholamines, one could speculate that the rapid relief of vasopressor dependency seen during PVT (Fig. 5a) might relax non-small vessels and thereby increase vessel density (TABLE 3; Fig. 4A, B).

A similar time course as seen for microcirculatory changes under PVT was described for the effect of venoarterial extracorporeal membrane oxygenation (VA-ECMO) on microcirculation in patients with refractory cardiogenic shock [29]: a sustained rise in MFI was seen as early as 2 h after starting VA-ECMO, while the density of small vessels remained nearly unchanged during the whole VA-ECMO treatment period of 48 h. MFI (median) rose from 2.1 before VA-ECMO to 2.5 (+ 19%) after 2 h of VA-ECMO, to 2.6 (+ 24%) after 4 h, to 2.8 (+ 33%) after 12 h, to 2.7 (29%) after 24 h and to 2.8 (+ 33%) after 48 h. The respective values from the MicrocircMODS study (Tables 1 and 3; Fig. 4) were as follows: before PVT (MTP 1): MFI 2.1; 2 h after initiation of PVT (MTP 3): MFI 2.5 (+ 19%); 4 h after initiation of PVT (MTP 5): MFI 2.6 (+ 24%); 24 h after initiation of PVT (MTP 9): MFI 2.7 (+ 29%); and 48 h after initiation of PVT (MTP 17): MFI 2.7 (+ 29%). Thus, in a quantitative manner, comparable increases in MFI by about 20–30% have been described for PVT and for VA-ECMO in the respective patient groups with similarly abnormal microcirculation.

### Physical Vascular Therapy and macrocirculation

Most ICU patients with MODS in the early phase require vasopressors to stabilise blood pressure. Therefore, it was surprising that even within the first 24 h after starting PVT, vasopressor use could be substantially reduced (Table 4 Part a; Fig. 5A). Despite tapering vasopressors, CI increased by 20% as early as 2 h after the first 8-min PVT on day 1 (Supplemental Table S1) and increased even more up to 40% after the four-day PVT period (Table 4 Part a; Fig. 5A). Accordingly, blood pressure stabilised with an increase in MAP from 71 mmHg before to 81 mmHg after the 4-day PVT period (Table 4 Part A; Fig. 5A). The increase in CI was mainly due to an increase in SVI (Table 4 Part a; Fig. 5A). This rise in SVI might probably be attributed to a reduction in afterload at the microcirculatory level triggered by PVT (see “SVRI” in Table 4 Part a; Fig. 5A; Supplemental Table S1). In an attempt to translate these findings to a prognostic message for our study patients with MODS of either cardiac or septic origin, we calculated the respective values for cardiac power index (CPI) and afterload-related cardiac performance (ACP) [17]: Before PVT, our study patients were characterized by a lowered CPI of 0.41 N × m^−2^ as well as by a lowered ACP of 70% of normal. Parallel to PVT, an increase in CPI from 0.41 to 0.53 N × m^−2^ (median; + 29%) and in ACP from 70 to 78% (median; + 11%) was observed (Table 4 Part a; Fig. 5A), both indicating a trend to improved outcome of the patients after the 4-day PVT period. None of these positive haemodynamic findings were observed in our small, similar-sized post hoc historical control group of matched pairs from the MOD*I*_f_Y trial during the initial four-day period: neither a rapid decline in vasopressor use nor an increase in CI, SVI, MAP, CPI, and ACP (Table 4 Part b; Fig. 5B; Supplemental Table S1). It is worth mentioning that study and historical control group had very similar median values for the prognostically relevant parameters APACHE II score (12 vs. 12; Table 2) and heart rate (92/min vs. 90/min; Table 4). Nevertheless, the authors are fully aware that using historical controls for comparison with study results is always related to numerous problems and therefore represents—at best—only a weak support.

It is further noteworthy that haemodynamic stabilisation during PVT coincided with a strong decrease in the APACHE II score by 7 points, from 32 points before PVT to 25 points after PVT, thereby indicating an improvement in survival probability. In contrast, the decrease in the APACHE II score by 4 points was considerably lower in the historical control group (Table 4 Part b).

Although vasopressors and inotropes are widely used in haemodynamically compromised ICU patients, there is insufficient evidence that these drugs are associated with reduced mortality [2, 30]. However, serious side effects of these substances, such as arrhythmias, lactic acidosis, worsening shock, and the development of multiple organ failure, may further worsen the prognosis of critically ill patients [2]. Therefore, it would make sense that new complementary concepts to treat haemodynamic impairment in MODS patients should not only stabilise cardiac output and blood pressure, but also enable a reduction or even abandonment of vasopressors and inotropes [31], as was observed for PVT in our MicrocircMODS study.

### Limitations of the MicrocircMODS study

Undoubtedly, this small, monocentric, one-arm safety and feasibility pilot study with 10 patients with MODS and post hoc historically matched pairs can only give a first impression of whether PVT might be able to influence microcirculation in a positive manner, thereby improving the haemodynamics of patients with MODS. Comparison of study results with a small historical control group is always related to pitfalls, excludes definitive answers and cannot replace comparison with a randomized control group.

The finding of a sustained stimulation of microcirculatory flow by PVT over the 4-day period as measured by MFI (Table 3; Fig. 4) has, of course, to be judged with great caution, taking into account the variable MFI course over time of MODS patients under standard care. In the MicroDAIMON study [3] with 97 critically ill patients, one-third of the patients showed an initial increment in MFI, one-third a reduction, and one-third no change. On the other hand, the abrupt increase in MFI by 24% immediately after the first PVT (Fig. 4 upper graph; Table 3) argues for a PVT effect and against a random effect. Finally, the improvement of both microcirculation (Table 3, Fig. 4) and macrocirculation (Table 4 Part A, Fig. 5A; Supplemental Table S1) may be in favour of some PVT-mediated regain of lost haemodynamic coherence.

As described in “Methods”, the concept of PVT is based on stimulation of impaired vasomotion and thereby improving microcirculation. Most experimental findings supporting this concept came from measurements of the amplitude–frequency spectrum of spontaneous arteriolar vasomotion in the subcutaneous tissue of probands carried out by a single research group [16, 25–27]. In the MicrocircMODS study, we were not able to measure vasomotion in our patients but had to restrict ourselves to the measurement of sublingual microcirculatory density and flow. Therefore, we cannot determine whether the observed improvement in microcirculatory function and global haemodynamics which parallel PVT is indeed due to PVT-regenerated vasomotion, or whether it is mediated by other mechanisms that improve microcirculation.

In summary, based on the experience of our MicrocircMODS study, PVT appears to be a safe and feasible complementary treatment for patients with MODS. With the SDF technique, augmentation of sublingual microcirculatory flow was measured during the four-day PVT period, which was accompanied by stabilisation of the macrocirculation, an increase in blood pressure and cardiac index, and early weaning from vasopressors. In view of the pilot nature of our non-randomized study, these findings are at best an incentive for a larger randomized trial to test the effect of PVT in patients with MODS. Because measurement of sublingual microcirculation by the SDF technique is not routinely available in the ICU [32–34], from a practical point of view, the most robust signals to be measured could be the weaning from vasopressors and the improvement in global haemodynamics (increase in cardiac output). Based on the comparison of vasopressor weaning in the MicrocircMODS group and the historical MOD*I*_f_Y control group (Table 4, Fig. 5A, B), a sample size of approximately 300 patients should be sufficient to show a significant difference in a randomised controlled trial. Considering the well-known detrimental effects of vasopressors, more rapid weaning from vasopressors in patients with MODS by complementary PVT, as shown in the MicrocircMODS study, could be helpful in improving the prognosis of patients with MODS.

### Electronic supplementary material

Below is the link to the electronic supplementary material.

Supplementary file1 (DOCX 30 KB)

## Data Availability

The datasets used and analysed during the current study are available from the corresponding author on reasonable requests.

## Appendix: independent data monitoring committee

Members of the committee: Prof. Dr. H. Ebelt, Internal Medicine Clinic II, Catholic Hospital “St Johann Nepomuk”, Erfurt, Germany; Prof. Dr. N. Cordes, National Center for Radiation Research in Oncology/Department of Radiotherapy and Radiation Oncology of the University Hospital and Medical Faculty of Carl Gustav Carus Technical University Dresden, Germany; Prof. Dr. C.E. Heyde, Department of Orthopedics, Trauma Surgery, and Plastic Surgery, University of Leipzig.
