# Supplementary material for: Treatment of patients with multiple organ dysfunction syndrome (MODS) with an electromagnetic field coupled to biorhythmically defined impulse configuration: the MicrocircMODS study
Source: Clin Res Cardiol. 2023 Sep 17;113(2):260–75. doi: 10.1007/s00392-023-02293-2 (PMC10850207; doi:10.1007/s00392-023-02293-2)
Supplement: Supplementary file 1 — Supplementary file1 (DOCX 30 KB) [file 392_2023_2293_MOESM1_ESM.docx]

SUPPLEMENTAL TABLE S 1.

**Physical Vascular Therapy BEMER^®^ (PVT) and global haemodynamics – baseline before and follow-up after the first 8-min-treatment episode on day 1.**

| **Indicator** | **Value** | **Baseline**  **before**  **first PVT**  **Measurement**  **Time Point 1** | **Follow Up**  **immediately**  **after first PVT**  **Measurement**  **Time Point 2** | **Follow up**  **2h**  **after first PVT**  **Measurement**  **Time Point 3** | **Follow up**  **4h**  **after first PVT**  **Measurement**  **Time Point 4** |
| --- | --- | --- | --- | --- | --- |
| **HR**  [1/min] | N  Absolute Median (IQR)  Rel. Median (IQR) | 8  92 (73-101)  100 | 8  96 (73-101)  104 | 9  94 (68-105)  102 | 9  103 (78-109)  112 |
| **CI**  [l x min^-1^ x m^-2^] | N  Absolute Median (IQR)  Rel. Median (IQR) | 7  2.5 2.1-3.1)  100 | 7  2.5 (2.1-3.1)  100 | 8  3.0 (2.3-3.4)  120 | 8  3.0 (2.3-3.4)  120 |
| **SVI**  [ml/m^2^] | N  Absolute Median (IQR)  Rel. Median (IQR) | 7  26 (20-44)  100 | 7  26 (20-44)  100 | 8  34 (27-40)  131 | 8  26 (25-36)  100 |
| **MAP**  [mmHg] | N  Absolute Median (IQR)  Rel. Median (IQR) | 8  71 (63-81)  100 | 8  66 (62-72)  93 | 9  73 (65-74)  103 | 9  74 (69-84)  104 |
| **SVRI**  [dyn x cm^-5^ x sxm^-2^] | N  Absolute Median (IQR)  Rel. Median (IQR) | 7  559 (266-1163)  100 | 7  479 (279-931)  86 | 8  385 (264-702)  69 | 8  501 (279-762)  90 |
| **CPI**  [W x m^-2^] | N  Absolute Median (IQR)  Rel. Median (IQR) | 7  0,41 (0,34-0,49)  100 | 7  0,35 (0,34-0,48)  85 | 8  0,48 (0,33-0,56)  117 | 8  0,46 (0,41-0,53)  112 |
| **ACP**  [%] | N  Absolute Median (IQR)  Rel. Median (IQR) | 7  69.75 (57.50-79.41)  100 | 7  62.66 (59.37-78.79)  90 | 8  72.51 (56.49-87.46)  104 | 8  72.14 (65.40-84.45)  103 |

For measurement time points (MTP) see TABLE 1. Values are given as absolute medians with interquartile ranges (IQR), as well as relative medians (%), with the median value on day 1 defined as 100 %. ACP = Afterload-related Cardiac Performance; CI = Cardiac Index; CPI = Cardiac Power Index; HR = Heart Rate; IQR = Interquartile Range; MAP = Mean Arterial Pressure; N = Number of patients measured; PVT = Physical Vascular Therapy BEMER^®^; SVI = Stroke Volume Index; SVRI = Systemic Vascular Resistance Index.

SUPPLEMENTAL TABLE S 2.

**Physical Vascular Therapy BEMER^®^ (PVT) and heart rate variability (HRV).**

| **Variable** | **Column 1**  **Normal Range** | **Column 2**  **MODS-Patients**  **(Historical Control)**  **(n = 85)** | **Column 3**  **MicrocircMODS**  **Day 1**  **Before PVT (MTP 1)**  **(n = 5)** | **Column 4**  **MicrocircMODS**  **Day 4 (after last PVT on day 4)**  **(MTP 32)**  **(n = 5)** |
| --- | --- | --- | --- | --- |
| **HRV-TD** |  |  |  |  |
| SDNN [ms] | 141 ± 39 | 57.7 ± 30.7 | 30.8 ± 24.2 | 25.8 ± 17.5 |
| SDANN [ms] | 127 ± 35 | 51.2 ± 29.7 | 28.0 ± 10.1 | 26.8 ± 12.1 |
| pNN50 [%] | 9 ± 7 | 4.8 ± 8.4 | 8.8 ± 12.2 | 4.0 ± 6.2 |
| rMSSD [ms] | 27 ± 12 | 26.9 ±26.6 | * | * |
| **HRV-FD** |  |  |  |  |
| LF [msec^2^] | 791 ± 563 | 129.3 ± 405.1 | 514.2 ± 687.7 | 71.8 ± 80.8 |
| HF [msec^2^] | 229 ± 282 | 112.3 ± 267.3 | 352.1 ± 549.4 | 43.1 ± 30.0 |
| VLF [msec^2^] | 1782 ± 965 | 191.3 ± 661.1 | 1548.8 ± 2788.0 | 653.2 ± 1082.6 |
| LF/HF | 4.61 ± 2.33 | 1.1 ± 0.9 | 2.12 ± 1.49 | 3.07 ± 3.21 |

Column 1 shows the normal range of HRV parameters and column 2 the values from 85 MODS patients (historical control), both coming from Schmidt et al [19]. Column 3 presents the HRV values from the MicrocircMODS patients obtained on day 1 before the first PVT, and column 4 the values taken on day 4 after the last PVT. All values are given as means ± standard deviations. HF = High Frequency Power; HRV-FD = Heart Rate Variability Frequency Domain; HRV-TD = Heart Rate Variability Time Domain; LF = Low Frequency Power; LF/HF = Ratio of Low versus High Frequency Power; MODS = Multiple Organ Dysfunction Syndrome; MTP = Measurement Time Point; pNN50 *=* Percent of Differences between Adjacent NN Intervals Exceeding 50 msec; PVT = Physical Vessel Therapy BEMER^®^; SDANN *= S*tandard Deviation of the Averages of NN Intervals in all 5-minute segments; SDNN = Standard Deviation of the NN Intervals; VLF = Very Low Frequency Power. *Not measured.
